# Colonization compatibility with *Bacillus altitudinis* confers soybean seed rot resistance

**DOI:** 10.1093/ismejo/wrae142

**Published:** 2024-07-29

**Authors:** Ping-Hu Wu, Hao-Xun Chang

**Affiliations:** Department of Plant Pathology and Microbiology, National Taiwan University, Taipei City 10617, Taiwan; Department of Plant Pathology and Microbiology, National Taiwan University, Taipei City 10617, Taiwan

**Keywords:** *Athelia rolfsii*, *Calonectria ilicicola*, *Fusarium oxysporum*, *Glycine max*, *Macrophomina phaseolina*, Microbiome, PacBio 16S rRNA gene full-length sequencing, Red crown rot, *Rhizoctonia solani*, *Sclerotinia sclerotiorum*

## Abstract

The plant microbiome and plant-associated bacteria are known to support plant health, but there are limited studies on seed and seedling microbiome to reveal how seed-associated bacteria may confer disease resistance. In this study, the application of antibiotics on soybean seedlings indicated that seed-associated bacteria were involved in the seed rot resistance against a soil-borne pathogen *Calonectria ilicicola*, but this resistance cannot be carried to withstand root rot. Using PacBio 16S rRNA gene full-length sequencing and microbiome analyses, 14 amplicon sequence variants (ASVs) including 2 ASVs matching to *Bacillus altitudinis* were found to be more abundant in the four most resistant varieties versus the four most susceptible varieties. Culture-dependent isolation obtained two *B. altitudinis* isolates that both exhibit antagonistic capability against six fungal pathogens. Application of *B. altitudinis* on the most resistant and susceptible soybean varieties revealed different colonization compatibility, and the seed rot resistance was restored in the five varieties showing higher bacterial colonization. Moreover, quantitative PCR confirmed the persistence of *B. altitudinis* on apical shoots till 21 days post-inoculation (dpi), but 9 dpi on roots of the resistant variety TN5. As for the susceptible variety HC, the persistence of *B. altitudinis* was only detected before 6 dpi on both shoots and roots. The short-term colonization of *B. altitudinis* on roots may explain the absence of root rot resistance. Collectively, this study advances the insight of *B. altitudinis* conferring soybean seed rot resistance and highlights the importance of considering bacterial compatibility with plant varieties and colonization persistence on plant tissues.

## Graphical Abstract

**Graphical Abstract:**
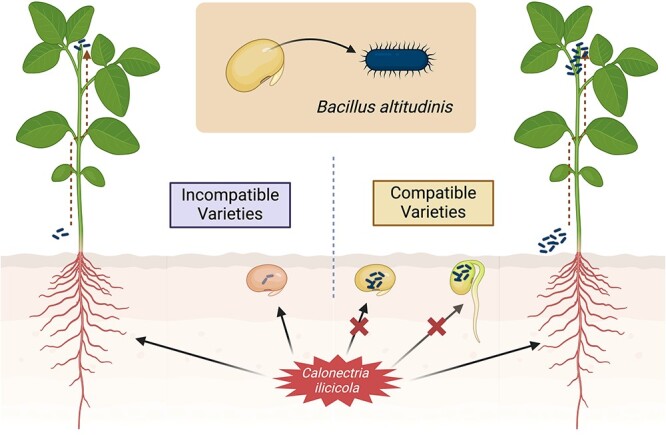

## Introduction

The plant microbiome constitutes a vast and complex community of microbes, playing a crucial role in supporting plant health, productivity, and resilience [1]. These microbes engage in various beneficial interactions with their hosts, such as nutrient acquisition, growth promotion [2, 3], and improving resistance to biotic and abiotic stresses [4–6]. As a result, the plant microbiome has received significant research attention due to its potential to sustain agriculture and the environment [7]. Although research over the past decade has predominantly concentrated on the phyllosphere and rhizosphere microbiomes, seed and seedling microbiome have been relatively overlooked despite its fundamental importance in plant growth and agricultural production.

The microbiome composition is shaped by factors such as plant species, genotypes, and environmental conditions. For example, seed endophytes are suggested to co-evolve with *Zea* species throughout domestication and geographical expansion [8]. In addition, domestication has been shown to decrease microbial diversity in wheat seeds, whereas domesticated rice exhibits a greater microbial diversity than their wild ancestors [9, 10]. Moreover, investigations indicate that the seed endophytic microbiome of rapeseed, pumpkin, and tomato can be influenced by host cultivar/genotype and environmental factors [11–13], whereas the seed epiphytic microbiome is primarily affected by environmental factors such as location [14]. Methodological variations, including the surface disinfestation procedure, can impact the detected species richness in different studies [15–17]. Nevertheless, there is a consensus across studies that seeds harbor fewer microbial species compared with other plant tissues [17]. This reduction in diversity raises interests to study how plants select the seed-associated microbes and their roles on plant health.

One of the initial perspectives on the seed-associated microbes stemmed from the recognition of seed-borne pathogens, which can cause diseases and significantly impact seedling health [18]. This understanding led to the widespread adoption of physical and chemical seed treatments, such as hot water soaking and seed coating, to eliminate seed-associated microbes in conventional farming. However, recent research is reshaping this perspective, unveiling a diverse range of beneficial bacteria and fungi in seeds. These seed-associated microbes are now recognized for playing crucial roles in seed germination, seedling development, and seedling protection from pathogens [19]. For instance, studies have demonstrated that rice and millet seeds treated with antibiotics, resulting in the absence of bacteria, exhibited slower germination processes [20, 21]. Similarly, maize and pearl millet seedlings treated with antibiotics showed reduced growth [22, 23]. Furthermore, endophytic bacteria such as *Bacillus*, *Pseudomonas*, and *Sphingomonas* have been identified as contributors to protect young seedling through the production of antimicrobial substances [5, 24] or the stimulation of plant defense responses [25, 26]. Some of these microbes can be vertically transmitted from parents to offspring plants, ensuring the continuity of a beneficial holobiont across generations [11, 17, 27, 28]. Accordingly, these findings suggest that seeds are the initial microbial reservoir, supporting the establishment of primary seedling holobiont. This concept not only reshapes the traditional approach of seed sterilization for disease management [29], but also underscores the potential of harnessing seed-associated microbes to develop sustainable disease management.

Soybean (*Glycine max*) holds significant agricultural importance worldwide. One of the primary challenges in soybean cultivation is the prevalence of seed-borne and soil-borne diseases, which can hinder seed germination through seed rot, root rot, and damping-off [30]. In the USA, these diseases together known as seedling diseases, accounting for 76% of soybean yield loss related to diseases from 2018 to 2020 [31]. Various fungal pathogens like *Athelia rolfsii*, *Fusarium oxysporum*, *Macrophomina phaseolina*, *Rhizoctonia solani*, and *Sclerotinia sclerotiorum* have become persistent endemic problems [32], and a recently emerging disease, red crown rot (RCR) caused by *Calonectria ilicicola*, has gained attention globally in recent years [33, 34]. Currently, seed coating with fungicides is commonly suggested to manage both endemic and emerging diseases [32, 35]. Alternatively, strategies such as plant resistance and beneficial microbes may offer more sustainable options for disease management. However, studies have pointed out that limited resistance in the global soybean germplasms to RCR, therefore, regional varieties should be evaluated to extend the search of resistant source [36, 37]. Regarding seed-associated bacteria of soybean, ~30 bacterial genera such as *Bacillus*, *Pantoea*, and *Sphingomonas* were identified from soybean seeds [38], and some of these seed-associated bacteria exhibited *in vitro* antagonistic activity against various soybean pathogens [39].

In this study, we found seed rot resistance but not root rot resistance against *C. ilicicola* among 16 local soybean varieties in Taiwan. We discovered and hypothesized the seed rot resistance may be attributed to seed-associated bacteria, rather than plant innate immunity. To verify the hypothesis, we employed antibiotic treatment to confirm the contribution of seed-associated bacteria, and utilized PacBio 16S rRNA gene full-length sequencing, which is a better technology than the 16S rRNA gene V3-V4 short read sequencing to uncover species-level resolution [40, 41] and explore the differential abundance of soybean seedling microbiome. We identified and characterized the colonization of *Bacillus altitudinis* on compatible soybean varieties is crucial for preventing seed rot, and the impersistent colonization of *B. altitudinis* on roots is the cause that the seed rot resistance cannot be carried to root rot resistance. Collectively, this study presents the instance of seed-associated *B. altitudinis*, exhibiting antagonistic capability depending on the bacterial density and persistence on soybean varieties and tissues.

## Materials and methods

### Preparation of fungal and plant materials

Fungal materials including *A. rolfsii* isolate a31, *C. ilicicola* isolate F018, *F. oxysporum* isolate R1031, *M. phaseolina* isolate 1-4-03, *R. solani* AG-7 isolate WDG070, *S. sclerotiorum* isolate 1980 were routinely cultured on potato dextrose agar (PDA) plates (HIMEDIA, India), and maintained on filter papers at −20°C or preserved in 20% glycerol at −80°C for long-term storage.

Upon experimental setup, soybean seeds were triply washed with tap water and then immersed in 1% NaOCl for 10 min, followed by repeatedly rinsing with sterile distilled H_2_O for five times. The completeness of surface disinfestation was assessed by spreading 100 μL aliquot of the last rinse on nutrient agar (NA) plates (HIMEDIA), and then incubated at 28°C for 5 days. Seeds were considered surface-disinfested and would be used in subsequent experiments if no microbial colony was formed on the NA plates. There are 16 varieties of soybean seeds used in this study (Table S1).

### Phenotyping soybean disease resistance to *C. ilicicola*

The seed rot assay was conducted according to Broders et al. [42] with slight modifications. In brief, conidia were collected from a 10-day-old *C. ilicicola* colony on ½-strength PDA (½PDA) and diluted to 2 conidia per microliter. A total of 100 μL of the diluted conidia (200 conidia) were spread on 1.5% water agar (WA) plates and incubated at 25°C in the dark for 2 days. Subsequently, eight surface-disinfested soybean seeds were placed on each WA plate and cultured at 25°C in the dark for 5 days. Surface-disinfested soybean seeds of each variety on the WA plates without conidia were included as the controls. Soybean seeds were considered dead for situations including no germination, the radicle length was <1 cm, or the cotyledon and radicle were fully colonized by mycelia. The seed mortality rate was represented by the number of dead seeds divided by the eight seeds in each WA plate. Each plate was counted as one biological replicate, and each experiment was constituted of three biological replicates. The experiment was repeated three times independently.

For the cotyledon rot assay, the severity of cotyledon rot was scored on a six-grade scale based on the lesion size and severity on the cotyledon: 0 = no lesion; 1 = lesion <25%; 2 = lesion less ranging from 26 to 50%; 3 = lesion less ranging from 51 to 75%; 4 = lesion over 75%; 5 = seeds with no germination or all cotyledon and radicle were colonized by mycelia. The scores of eight seedlings were averaged to represent one biological replicate; in other words, each plate was considered as one biological replicate. Each experiment was constituted of three biological replicates. The experiment was repeated three times independently. Finally, the seed rot severity was calculated by standardizing the seed mortality and the cotyledon rot to range from 0 to 1 and averaging these two indices.

For the root rot assay, the fungal inoculum was freshly prepared according to the previous methods [35], and 15 mL of the fungal inoculum or control inoculum was mixed with commercial potting soils (T033, Garden Castle Ltd, Taiwan) in 500 mL pots. Each pot contained four soybean seeds, and these pots were placed in a greenhouse at 25°C in a 16 h–8 h light–dark cycle with daily irrigation. After 21 days, soybean seedlings were collected to separate roots from soils by gently rinsing with tap water. The disease severity was visually scored using a six-grade scale: 0 = no symptom; 1 = small brown necrotic lesions on the primary root; 2 = brown necrotic lesions extending over the primary root and some lateral roots; 3 = over half root lost and rotted with brown necrosis on the subterranean stem; 4 = almost all roots lost and rotted; and 5 = dead seedlings [36]. The scores of four seedlings were averaged to represent one biological replicate; in other words, each pot was considered as one biological replicate. The experiment included 8 varieties × 2 treatments (inoculation or not) in a complete randomized design, and each factorial combination included 3 biological replicates (pots). The experiment was repeated three times independently.

### Elimination of seed-associated bacteria by antibiotics

To assess the role of seed-associated bacteria in the seed rot resistance, soybean seeds were firstly surface-disinfested as abovementioned method, and then soaked in a solution containing ampicillin (100 μg/mL), rifampicin (50 μg/mL), and streptomycin (100 μg/mL) for 16 h (Fig. S1). The control group was soaked in sterile distilled H_2_O or DMSO solution for the same period. Following treatment, the seeds were rinsed seven times with sterile distilled H_2_O to remove antibiotics residue. To ensure the completeness of eliminating seed-associated bacteria, the seeds were ground using a mortar and pestle in 2-fold volume (v/w) of phosphate-buffered saline (PBS) buffer, and 100 μL of the grinding aliquot was spread onto tryptic soy agar (TSA) plates (STBIO MEDIA). After incubating the TSA plates at 28°C for 5 days, the elimination of seed-associated bacteria was considered successful if no colony was observed.

### PacBio 16S rRNA gene full-length sequencing and microbiome analyses

DNA was extracted from 5 day-post-germination soybean seeds using the CTAB method. Each biological replicate was a pool of eight seeds within a Petri plate, and five biological replicates were included from each variety. Based on the seed rot resistance, the 4 most resistant and the 4 most susceptible soybean varieties were included, therefore, a total of 40 DNA samples were subjected to PacBio 16S rRNA gene full-length sequencing. The bacterial 16S rRNA gene was amplified by the universal primers 27f and 1492r (Table S2), sourced from the PacBio library preparation kit. To minimize the amplification of host DNA, the PNA blocker targeting soybean chloroplast DNA [43] was incorporated at a final concentration of 2.5 pmole, and the LNA blockers were added to target soybean mitochondria DNA [44] at a final concentration of 5 pmole (2.5 pmole for each direction). The sequencing libraries were prepared according to the workflow of the PacBio SMRTbell kit, and sequencing was performed on the PacBio Sequel IIe platform with 10 h movie collection time.

The raw sequencing files were filtered, trimmed, and de-replicated using the PacBio single-molecule real-time link software to generate circular consensus sequencing reads. The R package “DADA2” v1.26 was utilized to denoise and construct amplicon sequence variants (ASVs) following the adjusted parameters [45]. The chimeras were removed before classifying the ASVs using the Bayesian classifier in DADA2 and the SILVA v138.1 database at 99% similarity [46]. The non-prokaryotic and unclassified ASVs were removed before finalizing the ASV table. To assign the taxonomy to each ASV, the sequences were aligned using BLAST+ to NCBI 16S rRNA gene database with an E-value at 10^−5^. The classification of each ASV was determined by the lowest E-value, followed by the highest Bit score, and then the highest identity. The BLAST results were processed by the R package “taxize” v0.9.1, resulting in a final taxonomy table. The ASV sequences were aligned using MAFFT [47], and a maximum likelihood phylogenetic tree was constructed with IQ-TREE2 with 1000 bootstrap replicates [48].

The ASV table, taxonomy table, and phylogenetic results were imported into the R package “phyloseq” v1.42, and four samples with fewer than 2000 reads were filtered [49]. ASVs with a mean of relative abundance below 0.01% across 36 samples or with occupancy below 5% (lower than 2 samples) were excluded from the subsequent analyses. To assess α-diversity indices, all samples were rarefied to the sample with the lowest sequencing depth using the R package “vegan” v2.64-4 and “picante” v1.8.2. To assess the β-diversity, ASVs were normalized by median sequencing depth before subjected to non-metric multidimensional scaling (NMDS) based on the Bray–Curtis distance. PERMANOVA was conducted using the “adonis2” function in “vegan” package with 999 permutations. The differential abundance of ASVs between the resistant and susceptible varieties was analyzed using the R package “DESeq2” v1.38.3 [50], and the significance was determined at the BH-adjusted *P* values at 0.05.

### Isolation of seed-associated bacteria and *in vitro* antagonistic assay against fungal pathogens

Surface-disinfested soybean seeds were germinated on 1.5% WA plates for 5 days before grinding using a mortar and a pestle. The ground aliquots were serial diluted till 10^−5^ folds using PBS buffer, and the 100 μL diluted aliquot was spread on soymilk agar (SA) plates, NA plates, Luria-Bertani agar (LA) plates, and 0.1% TSA plates. These plates were incubated at 28°C for 7 days, with three plates for each dilution fold. After incubation, colonies were differentiated and selected based on their morphological features. The selected colonies were subsequently single colony purified using the streak plate technique. Finally, the purified colonies were preserved in 25% glycerol at −80°C for a long-term storage.

The seed-associated bacteria were tested for their antifungal activity against fungal pathogens in the dual culture assay on TSA plates. A mycelial plug (5 mm in diameter) from the actively growing edge of each fungal species was placed on the center of a medium plate. The bacteria were cultured in tryptic soy broth (TSB) for 16 h and adjusted to OD_600_ value of 1. Subsequently, 2 μL of the bacterial aliquot was placed 3 cm from the mycelial plug on both sides of a TSA plate. This method tested fungal pathogens under varying conditions of temperature and time. Specifically, *A. rolfsii* and *C. ilicicola* were measured at 7 days post-inoculation (dpi), *M. phaseolina* and *S. sclerotiorum* at 5 dpi, and *R. solani* at 2 dpi. All fungi were cultured at 28°C in the dark, except for *S. sclerotiorum* that was cultured at 25°C. The inhibition rate was calculated by:


$$ \left(1-\frac{\mathrm{The}\ \mathrm{colony}\ \mathrm{diameter}\ \mathrm{of}\ \mathrm{the}\ \mathrm{dual}\ \mathrm{culture}\ \mathrm{plate}}{\mathrm{The}\ \mathrm{colony}\ \mathrm{diameter}\ \mathrm{of}\ \mathrm{the}\ \mathrm{control}\ \mathrm{plate}}\right)\times 100\% $$

For identifying the species of bacterial isolate TN5S8 and TN3S3, the genomic DNA were extracted using the Presto gDNA Bacteria Advanced Kit (Geneaid Biotech Ltd, Taiwan), and subjected to PCR using the 27F/1492R for 16S rRNA gene and UP1/2r for *gyrB* gene [51] (Table S2). The amplicons were submitted for Sanger sequencing (Genomics, Taiwan), and sequencing results were subjected to BLAST search in the NCBI database the alignment with ASVs, and phylogenetic analysis.

### Colonization of *B. altitudinis* TN5S8 on different soybean varieties

Antibiotics-treated soybean seeds were soaked for 16 h in the freshly prepared aliquot of *B. altitudinis* TN5S8 (hereafter abbreviated as TN5S8). Meanwhile, the control group was soaked in PBS buffer. The inoculant was prepared from a 24-hour-old bacterial culture in TSB, pelletized by centrifugation at 14 000 g for 3 min, before being resuspended and adjusted to a concentration of 10^7^ CFU/mL using PBS buffer. The treated seeds were air-dried in a laminar flow hood. Seed rot resistance was evaluated using the plate assay method abovementioned. The colonization efficiency of TN5S8 on each soybean variety was assessed by re-isolating TN5S8 from seeds using the method aforementioned. The colony numbers per gram of seeds were assessed by:


$$ \frac{\mathrm{Noumber}\ \mathrm{of}\ \mathrm{colonies}}{\mathrm{Dilution}\ \mathrm{rates}\times \mathrm{seeds}\ \mathrm{weight}\ \left(\mathrm{g}\right)} $$

To study the impact of TN5S8 on seed germination, two different treatments were tested, including the surface-disinfested seeds + TN5S8, and the surface-disinfested seeds + cell-free culture filtrate of TN5S8. The cell-free culture filtrate was prepared from a 3-day-old bacterial culture in TSB, centrifuging at 14 000 g for 3 min and filtering the supernatant through a 0.2 μm Millex filter (Merck KGaA, Germany). The impact of TN5S8 on seed germination was assessed by germination rate at 5 dpi.

### Quantitative PCR detection of *B. altitudinis* TN5S8

Soybean seeds of the resistant variety TN5 and the susceptible variety HC were surface-disinfested and inoculated with TN5S8, and then planted in pots containing a mixture of peat and perlite at a ratio of 4:1 in the greenhouse using the methods mentioned above. Plant tissue samples were collected at 5 timepoints. The cotyledons and epicotyls were collected for samples at 3 dpi, and the apical shoot and the first node were sampled for samples from 6 dpi onwards. The root samples were washed with sterile water to remove soil, and the taproots were collected for DNA extraction. Each biological replicate consisted of tissues from four plants in a pot; in other words, each pot was considered as the biological replicate, and there were five biological replicates obtained for each time point. The experiment was repeated twice independently.

Approximately 350 mg of plant tissues were homogenized in liquid nitrogen with a mortar and postal, and DNA was extracted using the CTAB method. Specific primers were designed to amplify a 106-bp fragment of the *gyrB* gene of *B. altitudinis* (Table S2). The quantitative PCR (qPCR) was performed on the CFX Connect Real-Time System (Bio-Rad Hercules, CA, USA) using genomic DNA, iQ SYBR green supermix kit (Bio-Rad), and 0.4 μM of each primer under the following thermocycling conditions: 95°C for 3 min; 40 cycles of 95°C for 10 s and 57°C for 30 s, with a melting curve processing from 60°C to 95°C for quality control. Genomic DNA of TN5S8 and soybean were serially diluted 10-fold to build standard curves for the mean Ct values against the DNA concentrations. Soybean actin gene Glyma.15G050200 was used as an internal control. Each biological replicate was technically repeated twice.

### Sequencing, assembly, and analyses of *B. altitudinis* TN5S8 genome

The genomic DNA extracted by Presto gDNA Bacteria Advanced Kit (Geneaid) was sent for the Oxford Nanopore Technologies (ONT) whole genome sequencing (BIOTOOLS Co, Ltd, Taiwan). DNA concentration, purity, and integrity were checked by the Qubit 4.0 fluorometer (Thermo Scientific) and the Qsep 100 system (Bioptic Inc, Taiwan). To construct ONT sequencing library, DNA fragments smaller than 10 kb were removed by Short Read Eliminator XS (PacBio). Subsequently, 1 µg of high molecular weight genomic DNA underwent end-repairing and dA-tailing using the KAPA End Repair and A-Tailing reagent (Roche), followed by the barcode and adapter ligation using the ONT Native Barcoding Kit 24 V14. The resulting DNA libraries were cleaned up to enrich fragments larger than 1 kb before being sequenced on the PromethION 24 device using the FLO-PRO114M flow cell (R10.4.1). In addition, the NEBNext DNA Library Prep Kit (New England Biolabs) was used to construct the sequencing library for the NovaSeq 6000 (Illumina) paired end 150-bp platform. Long-read sequences generated by PromethION were processed using Guppy’s Super-accurate basecalling 400 bps model. Reads with an average quality score above Q10 were assembled using Flye [52]. The Flye contigs were further polished with Medaka (https://github.com/nanoporetech/medaka), and a final sequence polishing was conducted using Homopolish [53]. Additionally, short-read sequences from the NovaSeq 6000 system were quality controlled using FastQC and Cutadapt. Filtered reads were then mapped to the contigs using BWA [54], and corrections were made with Pilon [55]. The corrected contigs were evaluated using QUAST [56] and BUSCO [57] to assess the genome quality. Gene locations were predicted using Prokka [58]. Annotation of the protein-coding sequence was conducted using the BLAST against the clusters of orthologous groups (COG) database. The final annotated chromosome was plotted using CIRCOS to show the gene locations, GC content, and COG annotation. Secondary metabolite biosynthesis-related gene clusters (BGCs) were predicted by the antiSMASH 7.0 [59], and 47 complete *B. altitudinis* genomes were obtained from NCBI database for a comparative analysis on the BGCs.

### Statistical analysis

All statistical analyses were conducted using the R environment 4.2.3. For the data analyses using the *t*-test, ANOVA, and Tukey’s HSD test, the normality was checked by the Shapiro test and Q-Q plot, and homoscedasticity was checked by the Levene’s test. For the data not fitting parametric assumptions, the Kruskal–Wallis test and Dunn’s test was applied. The *P* values of the Tukey’s HSD test and Dunn’s test were adjusted by the BH method for multiple comparisons. The significance of the statistical analysis was determined by α at 0.05.

## Results

### Phenotyping soybean disease resistance to *C. ilicicola*

**Figure 1:**
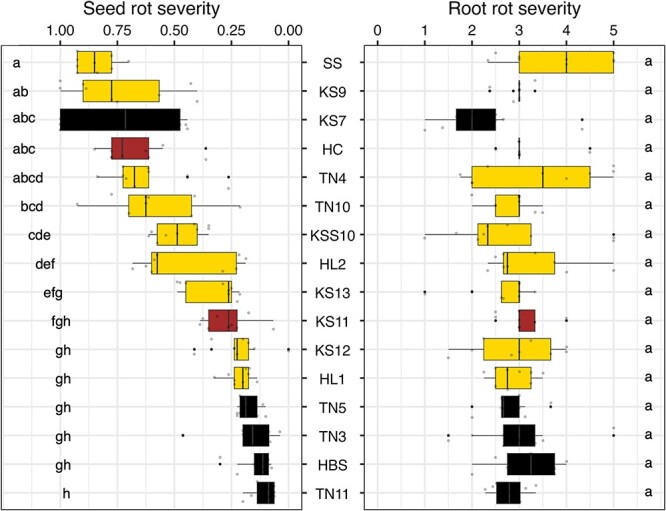
**Seed rot resistance and root rot resistance of 16 soybean varieties to *C. ilicicola*.** Seed rot severity was calculated by averaging the seed mortality rate and the cotyledon rot indices. Root rot severity was determined by the pot assay. The colors indicate the color of soybean seed coat. The Kruskal–Wallis and the Dunn’s test were used to determine significant difference at α = 0.05. There were three biological replicates (Petri plates or pots) for each variety and the experiment was repeated three times (*n* = 9).

In assessing soybean resistance to *C. ilicicol*a, significant differences were observed for both seed mortality (*P* < 0.001) and cotyledon rot (*P* < 0.001) across 16 local varieties of Taiwan. Soybean variety SS exhibited the highest levels of seed mortality rate and cotyledon rot score, whereas TN5 displayed the lowest seed mortality rate and TN11 displayed the lowest cotyledon rot score (Table S3). By averaging the seed mortality rate and the cotyledon rot score to obtain the seed rot severity, the results showed that TN11, HBS, TN3, and TN5 were the four most resistant varieties, whereas SS, KS9, KS7, and HC were identified as the four most susceptible varieties (Fig. 1).

**Figure 2:**
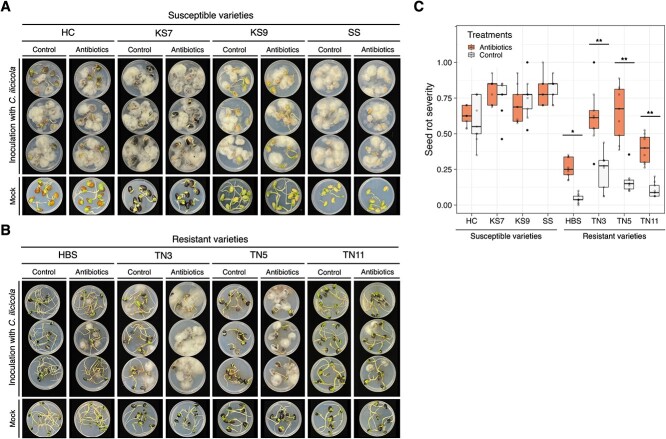
**Seed rot assay using the antibiotics-treated seeds reveals that seed-associated bacteria confer the seed rot resistance**. The antibiotics included ampicillin, rifampicin, and streptomycin. The control was treated with ddH_2_O. (A) The four most susceptible soybean varieties showed no difference between the control and antibiotic treatment. (B) The four most resistant soybean varieties showed significant difference in seed rot between the control and antibiotic treatment. Both seed mortality rate and cotyledon rot score were increased for the antibiotics-treated seeds, and the four initially resistant varieties became susceptible. (C) Seed rot severity. There were three biological replicates (Petri plates) for each factorial combination of variety and treatment, and the experiment was repeated three times (*n* = 9). The asterisks indicate significance based on the Tukey’s HSD test (^*^: *P* < 0.01, ^*^^*^: *P* < 0.001).

In contrast, no significant difference (*P* = 0.293) was observed for root rot resistance across the same 16 varieties (Fig. 1). These findings suggested that the soybean resistance to only seed rot, but not root rot, may not be solely determined by soybean innate immunity. It is possible that seed-associated bacteria play a role in seed rot resistance to *C. ilicicola*. Using antibiotics-treated seeds of the four most resistant and the four most susceptible soybean varieties, the susceptibility of the four susceptible varieties remained unchanged (Fig. 2A), but the four resistant varieties became susceptible (Fig. 2B, C). As the antibiotic treatment did not affect seed germination in the control groups and did not impact the growth of *C. ilicicola*, the increased seed rot severity in these four resistant varieties (TN11, HBS, TN3, and TN5) may be attributed to the elimination of seed-associated bacteria.

### PacBio 16S rRNA gene full-length sequencing and microbiome analyses

A total of 803 220 PacBio 16S rRNA gene full-length raw reads were acquired. Following quality controls and the exclusion of chloroplast, mitochondrial, and non-characterized sequences, 588 419 reads were retained (Table S4). Despite the inclusion of PCR blockers, two varieties (KS7 and KS9) still exhibited interference from Plant DNA (Fig. S2A). Consequently, two samples with fewer than 2000 reads from each of the KS7 and KS9 varieties were excluded from subsequent analyses. For the remaining 36 samples, rarefaction curves indicated satisfactory sampling depth, as all curves reached saturation status (Fig. S2B). After quality control, 145 ASVs were identified in the 36 samples, and the BLAST results showed an identity range of 85.33 to 100% for these ASVs according to the NCBI reference taxa (Table S5). Among them, 114 ASVs displayed identities greater than 99% to the reference taxa (Fig. S2C). Therefore, the microbiota obtained from PacBio 16S rRNA gene full-length sequences yielded a high-quality taxonomic profile at the species level for downstream analyses.

**Figure 3:**
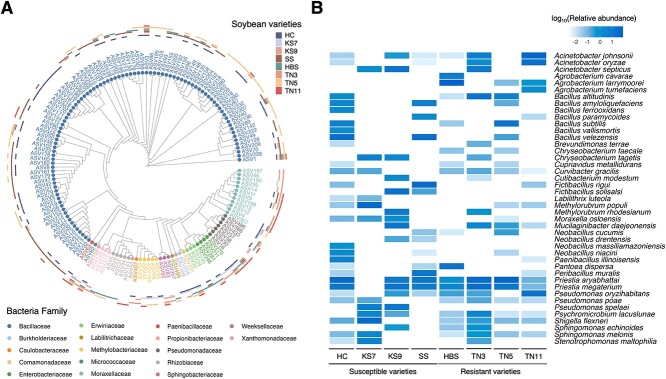
**PacBio 16S rRNA gene full-length analyses to identify the seed-associated bacteria of the eight soybean varieties.** (A) Maximum likelihood phylogenetic tree of the 145 ASVs the presence in each soybean variety. (B) Heatmap of log_10_(relative abundance) for the bacterial species in each soybean variety. There were eight seeds per plate and five Petri plates for each variety (*n* = 5).

The taxonomic profile unveiled four bacterial phyla associated with soybean, with the predominant taxa being *Bacillota* (56.4%), *Pseudomonadota* (41.0%), *Bacteroidota* (1.9%), and *Actinomycetota* (0.7%). At the family level, *Bacillaceae* emerged as the most abundant family (55.7%), followed by *Moraxellaceae* (15.0%) and *Rhizobiaceae* (11.7%) (Fig. 3A, Table S5). At the species level, the 145 ASVs were attributed to 44 bacterial species. Two bacterial species, *Priestia aryabhattai* and *Priestia megaterium*, were identified in seven varieties except for KS7. Seven bacterial species were present in six varieties (Fig. 3B, Fig. S3A). Additionally, 29 bacterial species were found in fewer than four varieties. The results indicated a considerable variability of the bacterial composition across the eight soybean varieties.

### α-Diversity, β-diversity, and differential abundance analyses

α-Diversity indices such as richness, Shannon diversity, and Pielou’s evenness, as well as β-diversity analysis using the NMDS based on the Bray–Curtis distance did not identify a clear separation between the four most resistant and the four most susceptible varieties (Fig. S3B-D). Indeed, PERMANOVA disclosed that the seed source contributed to 39.7% (*P* = 0.001) and soybean variety contributed to 6.8% (*P* = 0.003) of the total variance in microbial composition (Table S6). However, the seed rot resistance still accounted for 7.1% of the total variance (*P* = 0.002), suggesting microbial differences between the resistant and susceptible varieties.

**Figure 4:**
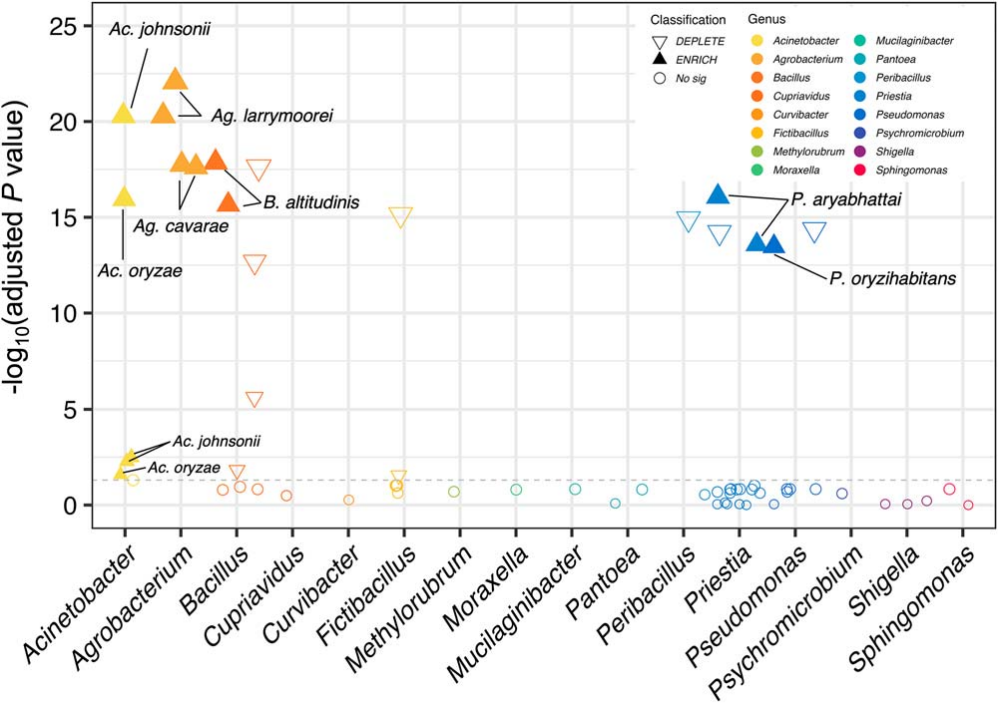
**Differential abundance analysis of the seed-associated bacteria between the resistant and susceptible varieties by DEseq2.** The Manhattan plots showing the ASVs, which are represented by circles or triangles. Whereas the circles are non-significant ASVs in the differential abundance analysis, the triangles are ASVs significantly enriched (filled) or depleted (empty) in the resistant varieties. The triangle size indicates the log_2_ fold change of the ASV. The y-axis indicates -log_10_(adjusted *P* value) and the x-axis represent the categorization of bacteria genus.

Differential abundance analysis identified 14 ASVs being more abundant in the four most resistant varieties versus the four most susceptible varieties (Fig. 4). These 14 ASVs were assigned to seven bacterial species, including *Acinetobacter johnsonii* ASV4, ASV11 and ASV12, *Acinetobacter oryzae* ASV13 and ASV14, *Agrobacterium cavarae* ASV31 and ASV34, *Agrobacterium larrymoorei* ASV2 and ASV5, *B. altitudinis* ASV20 and ASV48, *P. aryabhattai* ASV29 and ASV49, *Pseudomonas oryzihabitans* ASV38. (Fig. 4).

In pairwise comparison of these resistant and susceptible soybean varieties, *A. cavarae*, *A. larrymoorei*, *P. aryabhattai* were found as the significant species in the resistant variety HBS versus the other four susceptible varieties. *Priestia aryabhattai* was found in the resistant variety TN3 versus the other four susceptible varieties. As for the resistant variety TN5, *B. altitudinis* and *P. aryabhattai* were found as the significant species. Lastly, *A. johnsonii*, *A. larrymoorei*, and *P. oryzihabitans *were found in the resistant variety TN11 (Fig. S4). Collectively, the pairwise differential abundance analyses suggested the likelihood that different seed-associated bacteria may be involved to confer the seed rot resistance to *C. ilicicola*.

### Identification of *B. altitudinis* to inhibit fungal pathogens

A total of 300 bacterial isolates were obtained from the seedlings of eight soybean varieties, and 93 isolates with distinct colony morphology were selected for the *in vitro* antagonistic assay against *C. ilicicola*. The results identified 29 bacterial isolates that could inhibit at least 20% of the mycelial growth of *C. ilicicola* (Table S7). Among these isolates, one from TN3 and one from TN5 displayed a clear inhibition zone. Molecular identification and phylogenetic analysis of 16S rRNA and *gyrB* gene sequences revealed that both isolates belonged to *B. altitudinis* (Fig. S5A-B). The 16S rRNA gene sequences of these two bacterial isolates (TN3S3 and TN5S8) exactly matched *B. altitudinis* ASV20 and exhibited a single nucleotide difference with ASV48 (Fig. S5C).

**Figure 5:**
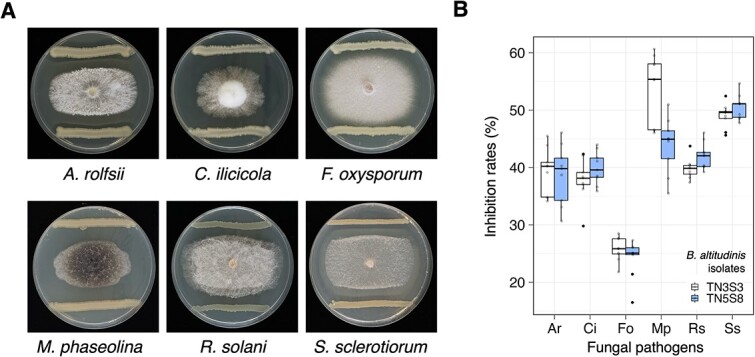
***In vitro* assay of *B. altitudinis* against six soil-borne fungal pathogens.** (A) Representative image of the dual culture assay using TN5S8 to antagonize six fungal pathogens. (B) The inhibition rate, which contains three biological replicates (Petri plates) and experiment was repeated three times (*n* = 9). Ar: *Athelia rolfsii*, Ci: *Calonectria ilicicola*, Fo: *Fusarium oxysporum*, Mp: *Macrophomina phaseolina*, Rs: *Rhizoctonia solani*, Ss: *Sclerotinia sclerotiorum.*

**Figure 6:**
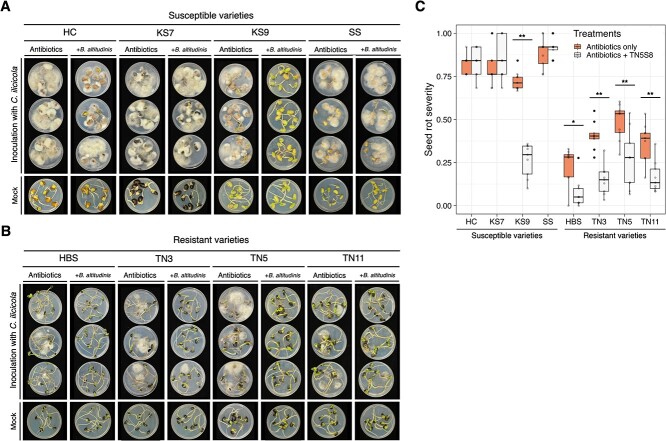
**Seed rot assay using the antibiotics-treated seeds inoculated with or without *B. altitudinis* TN5S8.** (A) The four most susceptible soybean varieties with three showed no difference with or without the inoculation of TN5S8. KS9 is the only variety being rescued by the inoculation of TN5S8. (B) The four most resistant soybean varieties showed significant reduction in the seed rot severity after the inoculation of TN5S8. (C) Seed rot severity. There were three biological replicates (Petri plates) for each factorial combination of variety and treatment, and the experiment was repeated three times (*n* = 9). The asterisks indicate significance based on the Tukey’s HSD test (^*^: *P* < 0.01, ^*^^*^: *P* < 0.001).

These two bacterial isolates (TN3S3 and TN5S8) exhibited antagonistic activity against other soil-borne pathogens, inhibiting the mycelial growth over 30% for *A. rolfsii*, *M. phaseolina*, *R. solani*, and *S. sclerotiorum*, and over 20% for *F. oxysporum* (Fig. 5). Using TN5S8 in the subsequent experiments, the results demonstrated that re-inoculating TN5S8 to the antibiotics-treated soybean seeds significantly mitigated seed rot caused by *C. ilicicola* across the four most resistant varieties and the susceptible variety KS9 (Fig. 6). These findings strongly suggested that *B. altitudinis* TN5S8 played a pivotal role in conferring the seed rot resistance against *C. ilicicola*.

The whole genome of *B. altitudinis* TN5S8 was sequenced to uncover the potential antifungal mechanisms. The genome of *B. altitudinis* TN5S8 comprises a 3747 068 bp circular chromosome with a GC content of 41.4% and 3771 coding sequences (Table S8, Fig. S6A). There were 10 secondary metabolite biosynthetic gene clusters identified in the TN5S8 genome (Fig. S6B), including two non-ribosomal peptide synthetase (NRPS) clusters with 85% similarity to the lichenysin gene cluster and 53% similarity to the fengycin gene cluster. Both lichenysin and fengycin were cyclic lipopeptides with antifungal properties [60, 61]. Additionally, a gene cluster encoding a siderophore was 60% similar to the schizokinen gene cluster, which may also contribute to the antifungal activity through nutrient competition [62]. Comparative analysis of secondary metabolite biosynthetic gene clusters across 48 *B. altitudinis* strains revealed that these three gene clusters were highly conserved within the *B. altitudinis* species (Fig. S7), suggesting the possible mechanism of antibiosis and nutrient competition for *B. altitudinis* to antagonize *C. ilicicola*.

### Colonization compatibility and persistence of *B. altitudinis* TN5S8 is a prerequisite to gain the seed rot resistance

**Figure 7:**
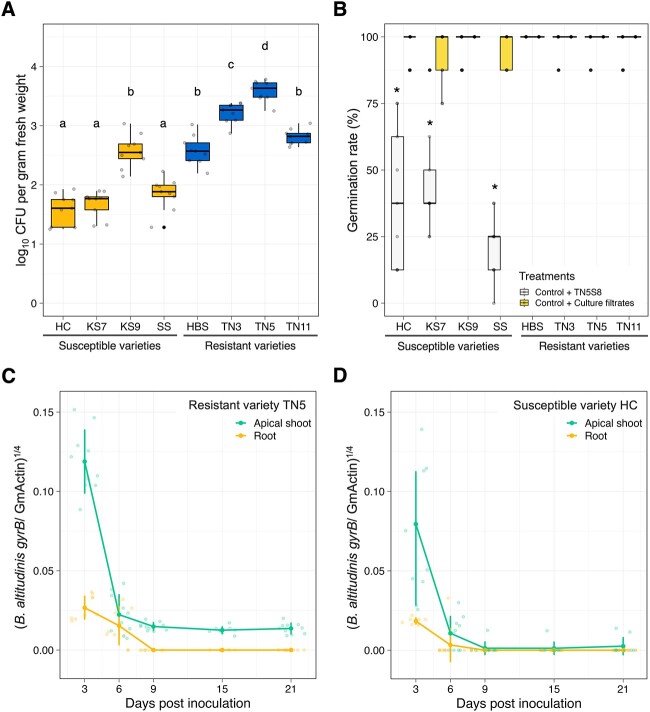
**Colonization compatibility and persistence of *B. altitudinis* TN5S8 on soybean varieties.** (A) The recovery of TN5S8 population on the antibiotic-treated seeds at 5 dpi. There were three biological replicates (Petri plates) for each variety, and the experiment was repeated three times (*n* = 9). ANOVA and the Tukey’s HSD test were used to determine the significance at α = 0.05. (B) The germination rates of the eight soybean varieties. The white bars indicate the inoculation of TN5S8 on the surfaced-disinfested seeds. The yellow bars indicate the application of TN5S8 culture filtrate on the surfaced-disinfested seeds. There were three biological replicates (Petri plates) for each variety, and the experiment was repeated three times (*n* = 9). The Kruskal–Wallis and the Dunn’s test were used to determine significant difference at α = 0.05. (C) qPCR to quantify TN5S8 on the apical shoots and roots of the resistant soybean variety TN5 at different timepoints. (D) qPCR to quantify TN5S8 on the apical shoots and roots of the susceptible soybean variety HC at different timepoints. The y axis indicates the transformed values of the absolute *gyrB* gene amount of *B. altitudinis* in soybean tissues represented by the absolute soybean actin gene amount. There were five biological replicates for each timepoint in each experiment. The experiment was repeat twice (*n* = 10).

The introduction of TN5S8 did not provide seed rot resistance for three susceptible varieties—HC, KS7, and SS. We postulated that the colonization compatibility between TN5S8 and soybean varieties might be a pivotal factor in gaining the seed rot resistance. Therefore, we re-isolated and quantified the TN5S8 population on the eight soybean varieties inoculated with TN5S8, and we observed a significantly higher density of TN5S8 in the four resistant varieties and the susceptible variety KS9, and a lower density for HC, KS7, and SS (Fig. 7A). The highest recovery of TN5S8 was observed from its original variety TN5. In addition, TN5S8 significantly impeded the seed germination of soybean varieties HC, KS7, and SS (Fig. 7B), and the non-germinated seeds of HC, KS7, and SS exhibited dark hue and soft rot. Furthermore, the germination reduction and diseased symptoms were not induced by the cell-free culture filtrate of TN5S8. These findings underscored the importance of colonization compatibility between TN5S8 and soybean varieties to confer the seed rot resistance.

Additionally, using qPCR to detect the presence of TN5S8 on the resistant variety TN5 and the susceptible variety HC, TN5S8 was found to persist on the apical shoot of soybean seedlings until 21 dpi, but it was not detected on the roots after 9 dpi on the resistant variety TN5 (Fig. 7C). In contrast, TN5S8 was rarely found on the apical shoots or roots of the susceptible variety HC after 6 dpi (Fig. 7D). Accordingly, the colonization compatibility of TN5S8 on seeds and the impersistent colonization on roots of the resistant TN5 provide an explanation for the seed rot resistance which cannot be carried to root rot.

## Discussion

Plant innate immunity has been recognized as the major underlying source of plant disease resistance, not only for soybean [63] but also for most important crops [64]. However, some studies have observed that fungal infection on different tissues such as seed, root, node, or leaf of the same plant genotype could result in different levels of resistance [65, 66]. For example, a study of soybean resistance to *Pythium* revealed the phenotypic correlation between seed rot and root rot was ranged from 0.1 to 0.17 [65]. Another study on the pea resistance to *S. sclerotiorum* reported the phenotypic correlation of nodal resistance and leaf resistance was only 0.19. Recently, it has been known that disease resistance can also be provided by the plant-associated microbes [67–69], therefore, the importance of considering the plant holobiont (including the plant host and the plant-associated microbes) as an entity has been increasingly recognized to uncover the mechanism of plant health [70].

Among the plant-associated microbes inhabiting on different tissues such as fruit, leaf, or roots, the seed-associated bacteria are the front line group in fighting against soil-borne diseases that mostly damage plants at the seedling stage [71]. For example, distinct bacterial compositions in the seeds of different oilseed rape cultivars were correlated with varying resistance levels to Verticillium wilt and *Plasmodiophora brassicae* [13, 72]. In another study, a seed endophytic bacterium *Sphingomonas melonis*, which can be vertically transmitted to the next generation of seeds, confers rice seedlings resistance against *Burkholderia plantarii* [5]. Similarly, *Bacillus velezensis* isolated from maize seeds and *Bacillus subtilis* found in millet seeds were shown to protect seedlings from *Fusarium* infection [22, 23]. In assessing 16 local soybean varieties, this study identified a discrete disease resistance, which is present only for seed rot, but not for root rot. The source of this discrete resistance may be something other than plant innate immunity, leading us to the hypothesis that seed-associated bacteria confer the seed rot resistance, which cannot be carried to the roots.

Based on the experiments using the antibiotics-treated seeds, the results confirmed that seed-associated bacteria were involved in the seed rot resistance of soybean. As previously reported that the α-diversity or co-occurrence network properties between the resistant and susceptible plants were different [72, 73] and may protect the resistant plants from pathogen [74], this study applied 16S rRNA gene full-length sequencing and microbiome analyses to compare the seedling microbiome between the resistant and susceptible soybean varieties. Nevertheless, there was no significant differences in the α-diversity or β-diversity. Instead, the differential abundance analysis discovered 14 ASVs that were significantly enriched in the four most resistant varieties, suggesting that a certain group of seed-associated bacteria may contribute to the seed rot resistance.

These 14 ASVs belong to the bacteria species such as *A. johnsonii*, *A. oryzae*, *A. larrymoorei*, *A. cavarae*, *B. altitudinis,* and *P. oryzihabitans. Acinetobacter johnsonii* has been previously isolated from soybeans [75], exhibiting antagonistic capabilities against soil-borne pathogens. In addition, *A. larrymoorei* and *P. oryzihabitans* have been reported as soybean endophytic bacteria, showing potential in the nitrogen fixation and phosphate solubilization [76, 77]. Moreover, *P. oryzihabitans* strains have antagonistic capability against pathogens such as *Acidovorax citrulli* in cucurbits and *Pythium* in cotton [78, 79]. Although literature suggested that these bacteria may play roles in the seed rot resistance, our culture-dependent isolation obtained two isolates, TN5S8 and TN3S3, which matched to another enriched ASVs identified as *B. altitudinis*. In our experiments, loss-of-function evidence through the antibiotic treatment and the gain-of-function evidence through the re-inoculation of *B. altitudinis* to soybean seeds confirmed the contribution of *B. altitudinis* in the seed rot resistance.

*Bacillus altitudinis* was first isolated from extreme UV-stressed air samples collected in the stratosphere [80]. It has been identified as an endophyte in various plants, including soybean [81] and others [82–89]. Several strains of *B. altitudinis* have shown biocontrol capabilities, such as cotton Verticillium wilt [82], grape downy mildew [85], kiwi fruit root-knot nematodes [89], soybean Phytophthora damping-off [81], and sweet potato black rot [87]. It has been shown that *B. altitudinis* can inhibit plant pathogens by producing antimicrobial lipopeptides lichenysin [60, 85] and inducing plant defense responses [81, 89]. Moreover, genome analysis also identified gene clusters similar to the fengycin and schizokinen gene clusters, and these compounds may be associated with the antagonistic ability [61, 62]. Our comparative genomics analyses have found that these secondary metabolite biosynthesis gene clusters are highly conserved in different strains of *B. altitudinis*, and more recently, *B. altitudinis* has been suggested to have an open pangenome with 42.7% genes characterized as accessory genes. These results indicated that *B. altitudinis* may tend to acquire new genes to enhance its antagonistic capability and ecological competitiveness [85].

We further observed this seed rot resistance depends on the colonization compatibility of TN5S8 on soybean varieties. The relationship between bacterial population and disease suppression echoes previous findings on the biocontrol efficacy of *Pseudomonas fluorescens* was proportional to their density [90], and the effective threshold ranges from 10^5^ to 10^6^ bacteria per gram of root against wheat take-all decline disease [90]. Similarly, the suppression of other Pythium root diseases in sugar beets also depended on the population density of the *Pseudomonas* [91, 92]. In rice, the abundance of *Sphingomonas* was observed to be lower in plants susceptible to seedling blight disease [5]. Specifically for the cases within the *Bacillus* genus, colonization and formation of biofilm on the phyllosphere or root surface is critical for the success of biocontrol [93]. On tomato, *Bacillus* strains with less colonization ability on the phyllosphere showed a reduced biocontrol ability against *Botrytis cinerea* [94]. Application of plant extracts such as pectin can enhance the *Bacillus amyloliquefaciens* population on tobacco roots and increase the biocontrol efficacy to tobacco bacterial wilt [95]. Mutation of *B. amyloliquefaciens abrB* gene, which is a negative transcription regulator of chemotaxis and biofilm formation, increased colonization and biocontrol capability against the cucumber Fusarium wilt [96]. In addition, the colonization of *B. subtilis* surfactin deletion mutant reduced 4 to 10-fold on the melon roots and leaves, which ended up losing the biocontrol efficacy [97]. However, it has not be reported whether the colonization of *Bacillus* can affect seed resistance, and our finding provided the evidence that the colonization compatibility of *B. altitudinis* on soybean seeds is an important factor to confer the seed rot resistance.

The prevalence of *B. altitudinis* was not uniform in all PacBio sequencing samples, meaning the ASVs assigned to *B. altitudinis* could be detected in some but not all samples of the four most resistant varieties (Fig. S3A). One possibility is that other seed-associated bacteria provide the seed rot resistance in samples where *B. altitudinis* was absent. Indeed, the differential abundance pointed out additional bacteria that may confer the seed rot resistance, with *B. altitudinis* was one of these bacteria. In other words, the seed rot resistance observed in other varieties where *B. altitudinis* was absent may be provided by other seed-associated bacteria that were not recovered from our culture-dependent isolation. Another possible cause may have been the nature of the seed bacterial community which was highly variable and stochastic according to the seed source and planting location (Fig. S8). The PERMANOVA results suggested a great proportion of microbiome variance was explained by the seed source. Moreover, because the seedling microbiome is assembled from the seed bacterial community, the process becomes a selection bottleneck to increase the variability of seedling microbiome.

Only a small fraction of seed taxa is transmitted to the seedlings [98, 99]. A recent study on oak showed that 63% of fungal taxa and 45% of bacterial taxa on the seeds can be transmitted to the seedlings [15]. Another study on tomato demonstrated that some seed-associated microbes such as *P. aryabhattai*, *Bacillus nakamurai*, *Ralstonia pickettii*, and *Stenotrophomonas maltophilia* could persist from seeds to seedlings for at least two generations [11]. However, even though the seed-associated bacteria can be transmitted to seedlings, their colonization on the shoots or roots may be different. A study on soybeans in an axenic environment demonstrated that the seed-transmitted bacterial ASVs dominant in the shoots can be rare or absent in the roots [100]. This report aligns with our observation on TN5S8, which was detected on the apical shoots for at least 21 dpi, but it could not be detected after 9 dpi on the root of the compatible and resistant variety TN5. As for the incompatible and susceptible variety HC, TN5S8 was rarely detected after 6 dpi, and the absence of TN5S8 on roots may result in no protection in the root rot assays.

In summary, this study identified that the seed-associated bacterium *B. altitudinis* could provide antagonistic capability to fungal pathogens, and *B. altitudinis* confers only the seed rot resistance in certain soybean varieties based on its colonization compatibility and persistence. The results highlight the future application of seed-associated bacteria in disease management to consider not only the antagonistic capability, but also the colonization compatibility and persistence on the plant varieties and tissues.

## Supplementary Material

Supplementary_material

## Data Availability

The raw microbiome sequencing data have been deposited in the NCBI Sequence Read Archive (SRA) under BioProject IDs PRJNA1099878. The whole genome sequence of *B. altitudinis* TN5S8 is available in GenBank with the accession number CP155530.1. Code to analyze microbiome data and generate figures and tables are located on GitHub at: https://github.com/wuphw/Soybean_seed_microbiome_resistance.
