# Supplementary figures and images for: Colonization compatibility with Bacillus altitudinis confers soybean seed rot resistance
Source: ISME J. 2024 Jul 29;18(1):wrae142. doi: 10.1093/ismejo/wrae142 (PMC11378728; doi:10.1093/ismejo/wrae142)

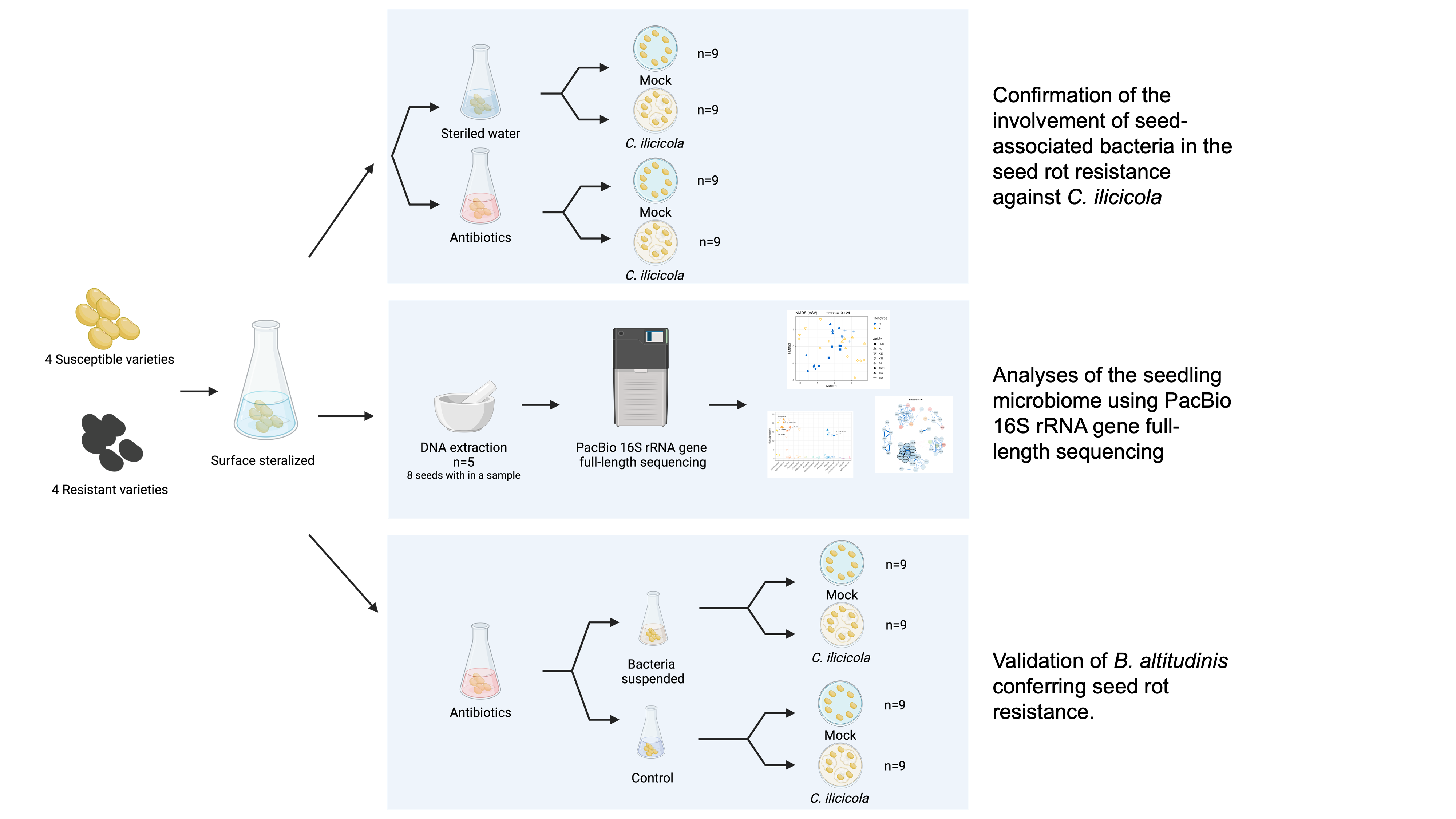

Supplement: Supplementary_material [file supplementary_material.zip › Fig. S1.tiff]

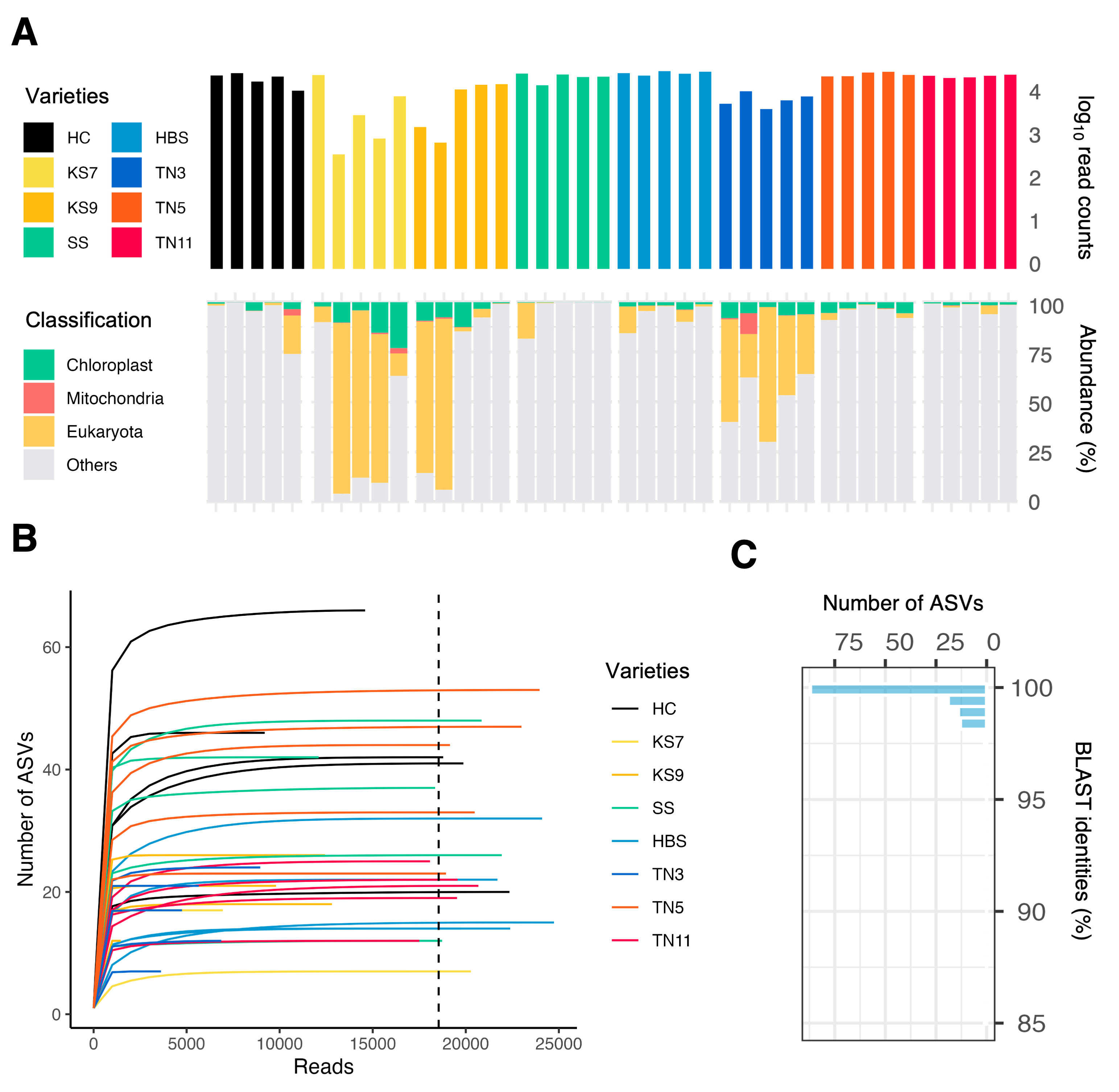

Supplement: Supplementary_material [file supplementary_material.zip › Fig. S2.tiff]

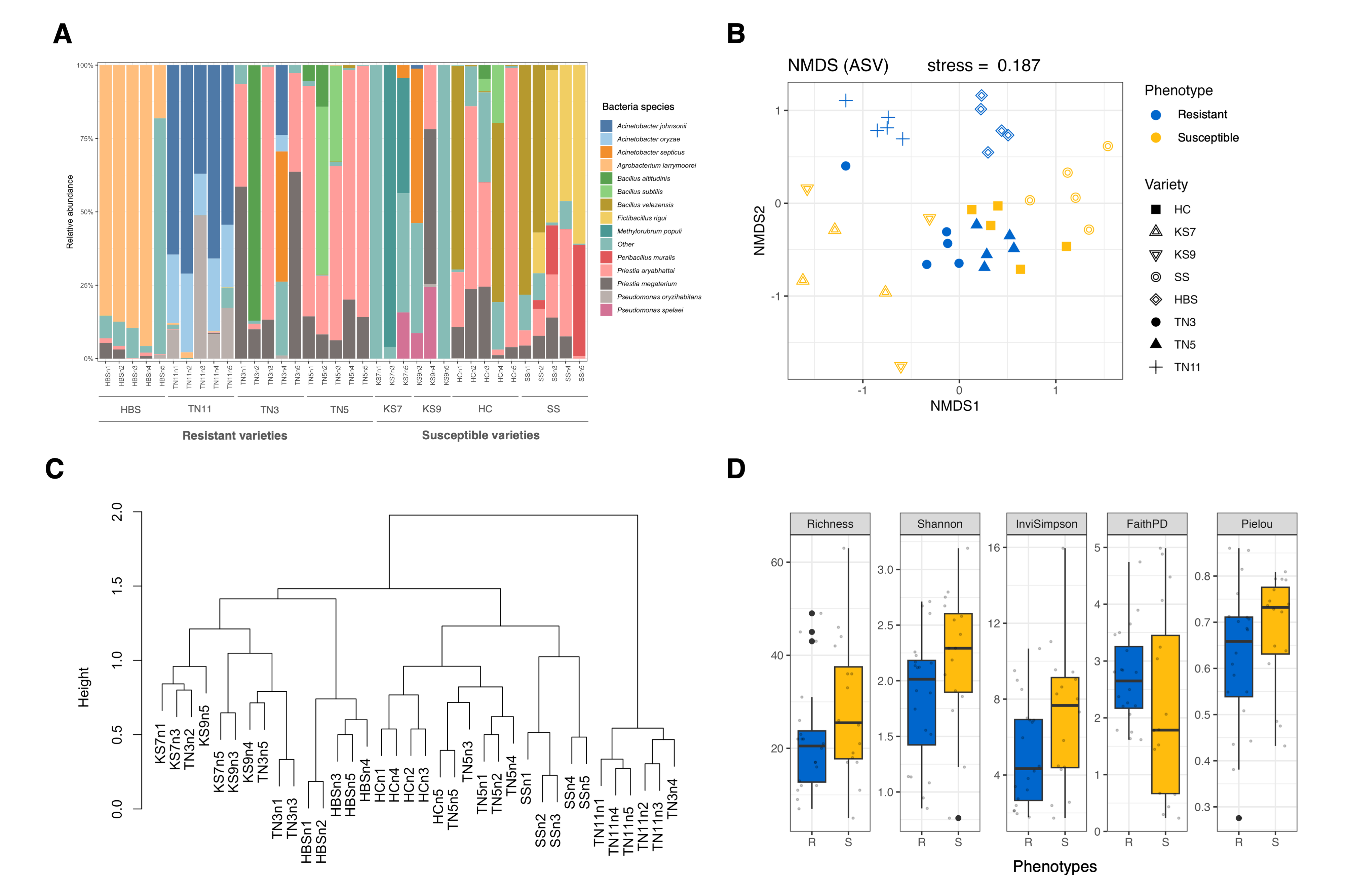

Supplement: Supplementary_material [file supplementary_material.zip › Fig. S3.tiff]

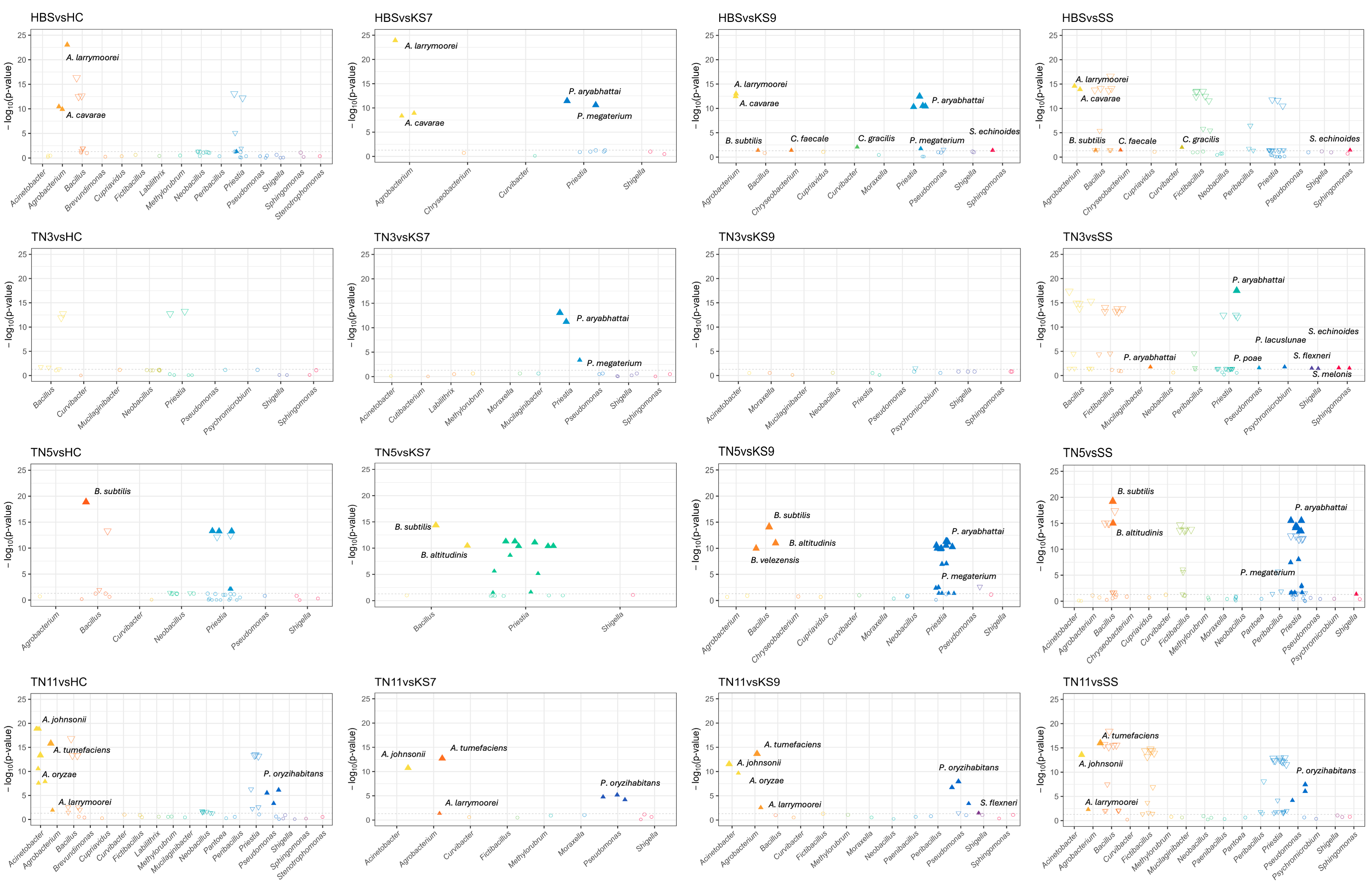

Supplement: Supplementary_material [file supplementary_material.zip › Fig. S4.tiff]

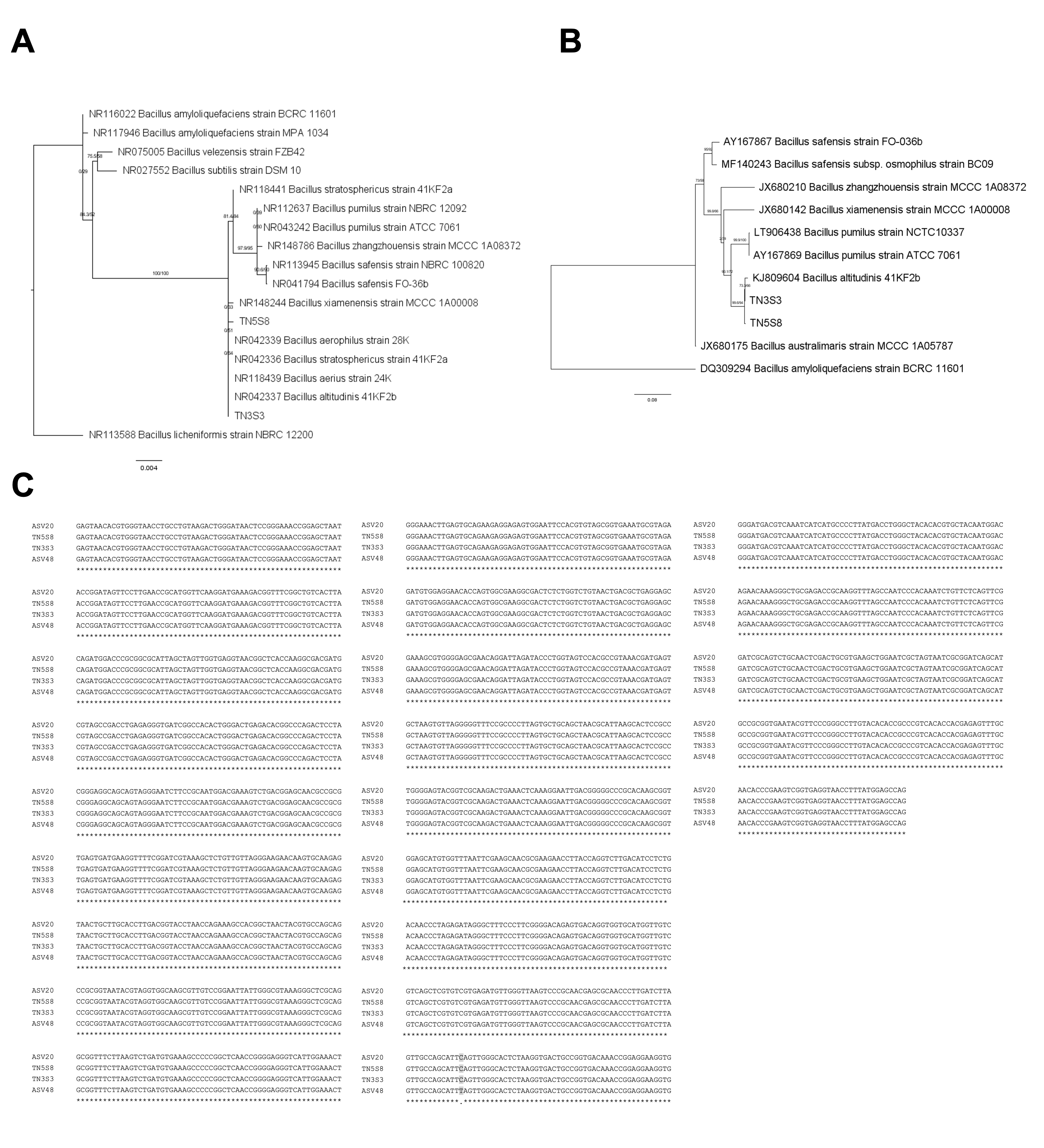

Supplement: Supplementary_material [file supplementary_material.zip › Fig. S5.tiff]

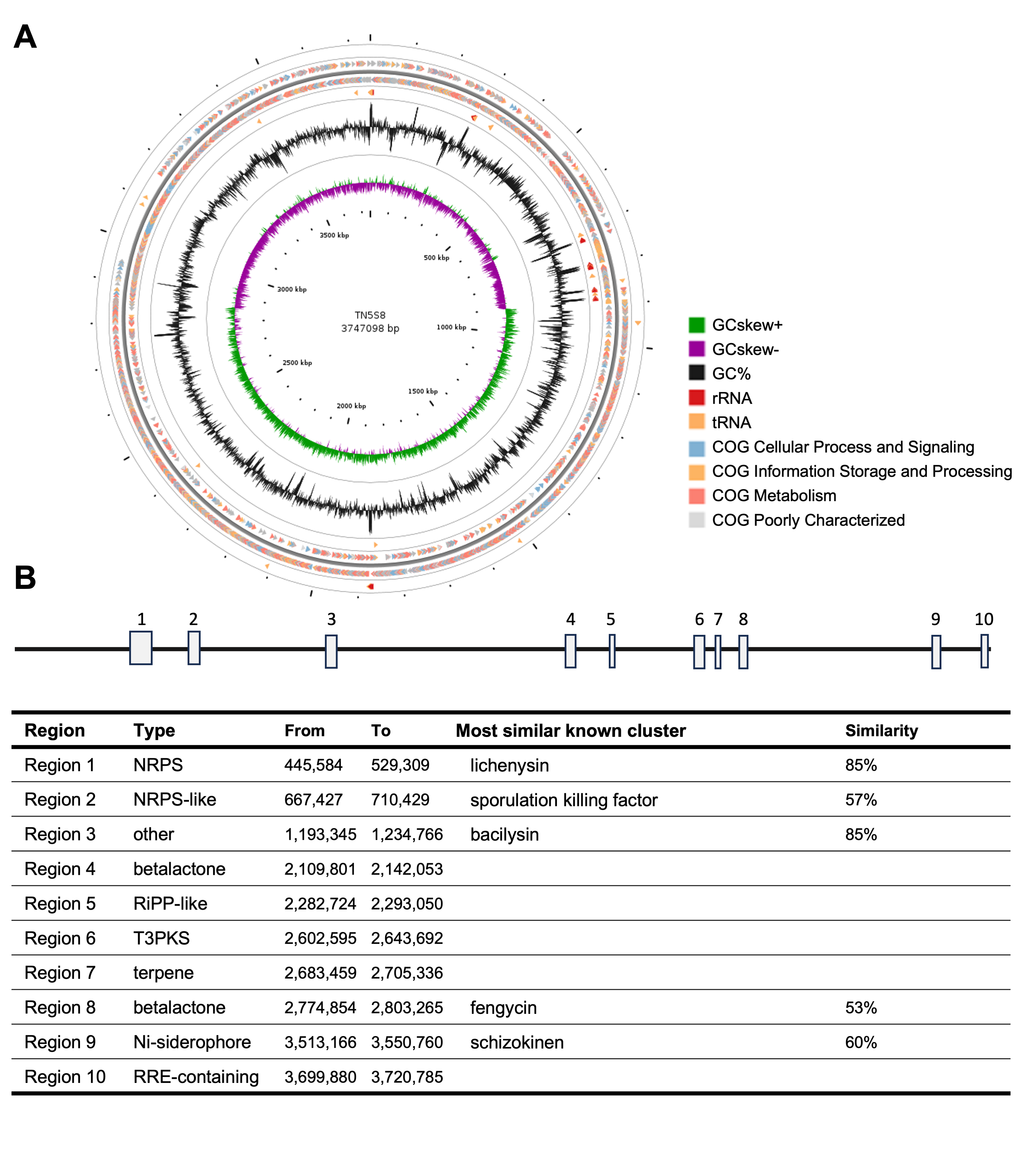

Supplement: Supplementary_material [file supplementary_material.zip › Fig. S6.tiff]

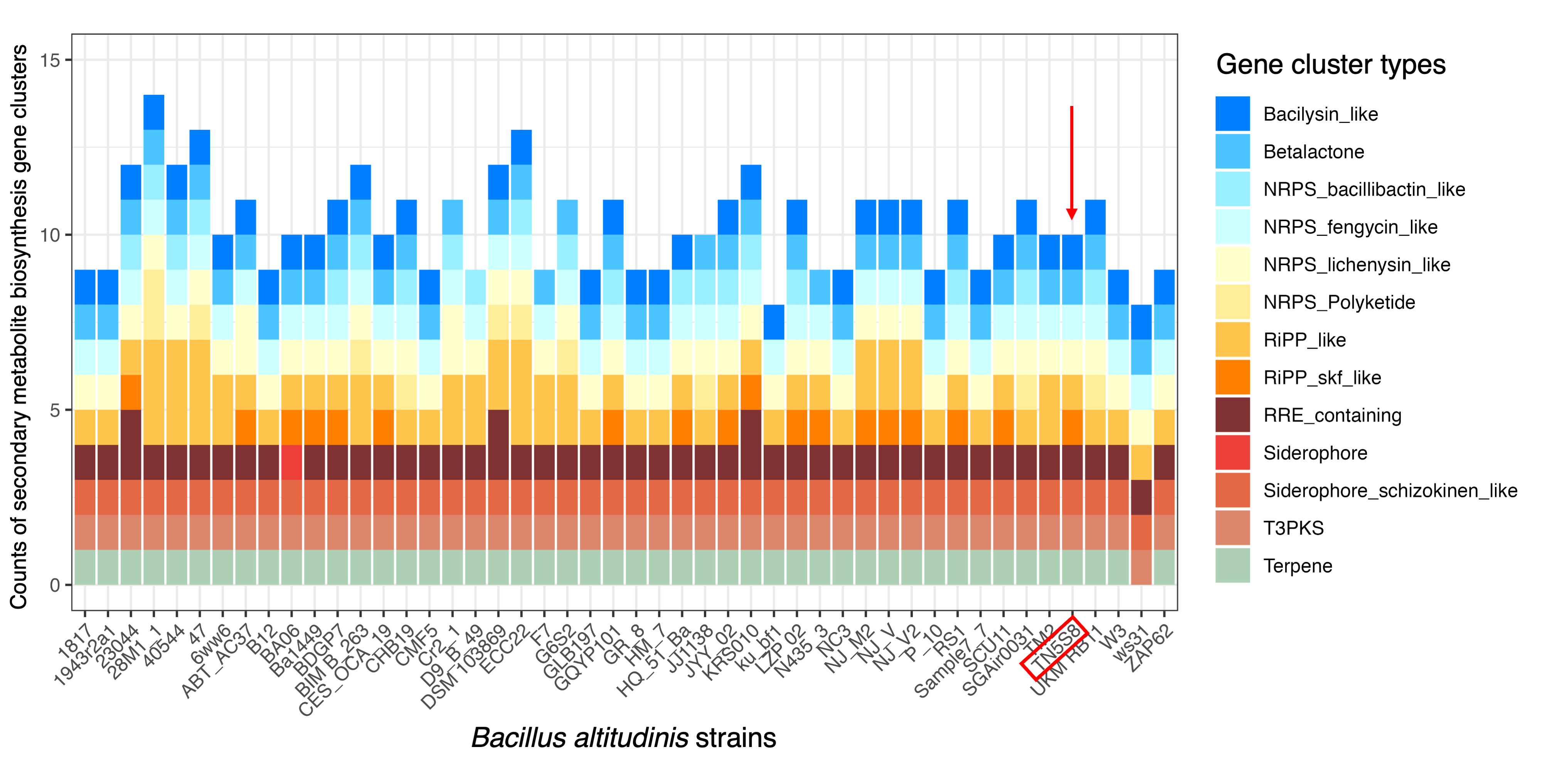

Supplement: Supplementary_material [file supplementary_material.zip › Fig. S7.tiff]

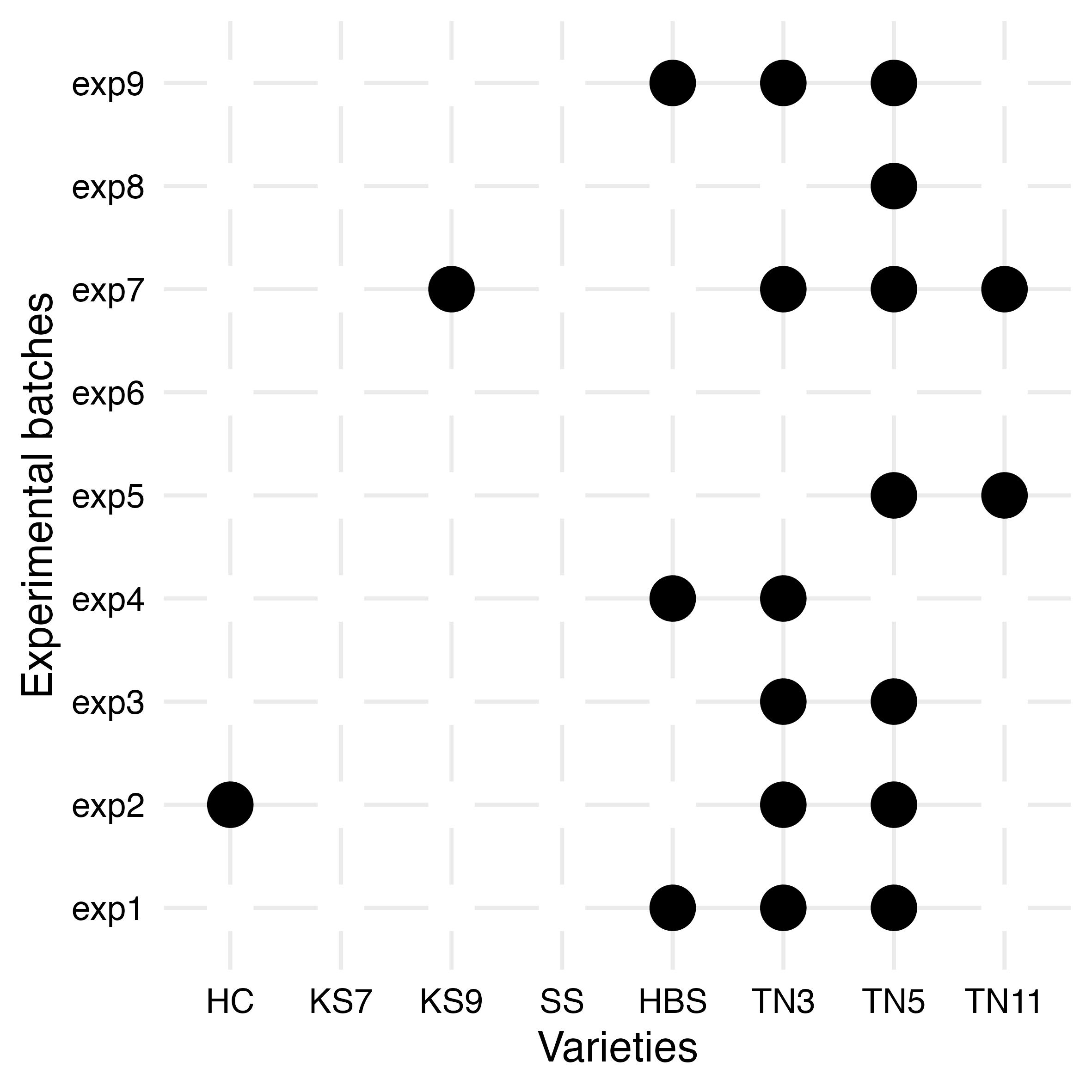

Supplement: Supplementary_material [file supplementary_material.zip › FigS8.jpg]
